# Non-syndromic enlarged vestibular aqueduct caused by novel compound mutations of the *SLC26A4* gene: a case report and literature review

**DOI:** 10.3389/fgene.2023.1240701

**Published:** 2023-09-07

**Authors:** Yunhua Huang, Linlin Li, Liqiu Pan, Xiaoting Ling, Chenghan Wang, Chaoyu Huang, Yifang Huang

**Affiliations:** ^1^ Department of Clinical Laboratory, The First Affiliated Hospital of Guangxi Medical University, Nanning, Guangxi, China; ^2^ Key Laboratory of Clinical Laboratory Medicine of Guangxi Department of Education, Guangxi Medical University, Nanning, Guangxi, China

**Keywords:** non-syndromic enlarged vestibular aqueduct, *SLC26A4*, compound mutations, sensorineural hearing loss, pendrin

## Abstract

Enlarged vestibular aqueduct is an autosomal genetic disease mainly caused by mutations in the *SLC26A4* gene and includes non-syndromic and syndromic types. This study aimed to identify genetic defects in a Chinese patient with non-syndromic enlarged vestibular aqueduct (NSEVA) and to investigate the impact of variants on the severity of non-syndromic enlarged vestibular aqueduct. A male patient with NSEVA, aged approximately 6 years, was recruited for this study. The clinical characteristics and results of auxiliary examinations, including laboratory and imaging examinations, were collected, and 127 common hereditary deafness genes were detected by chip capture high-throughput sequencing. Protein structure predictions, the potential impact of mutations, and multiple sequence alignments were analyzed *in silico*. Compound heterozygote mutations c.1523_1528delinsAC (p.Thr508Asnfs*3) and c.422T>C (p.Phe141Ser) in the *SLC26A4* gene were identified. The novel frameshift mutation c.1523_1528delinsAC produces a severely truncated pendrin protein, and c.422T>C has been suggested to be a disease-causing mutation. Therefore, this study demonstrates that the novel mutation c.1523_1528delinsAC in compound heterozygosity with c.422T>C in the *SLC26A4* gene is likely to be the cause of NSEVA. Cochlear implants are the preferred treatment modality for patients with NSEVA and severe-to-profound sensorineural hearing loss Genetic counseling and prenatal diagnosis are essential for early diagnosis. These findings expand the mutational spectrum of *SLC26A4* and improve our understanding of the molecular mechanisms underlying NSEVA.

## 1 Introduction

Enlarged vestibular aqueduct (EVA) is an autosomal recessive disease characterized by severe-to-profound sensorineural hearing loss (SNHL) and widening of the vestibular aqueduct (Azaiez et al., 2007; Zhang et al., 2016). EVA is one of the most common congenital inner ear defects, and early diagnosis is important for timely intervention. EVA results from mutations in the *SLC26A4* gene, including Pendred’s syndrome (PS) and non-syndromic EVA (NSEVA). Notably, some patients with EVA have only one mutation or lack detectable mutations. Patients with different mutations also exhibit high phenotypic heterogeneity. High intrafamily phenotypic variability occurs among individuals with PS, and the typical goiter phenotype of PS is variably penetrant; some patients may not display symptoms until adolescence (Rehman et al., 2017). Therefore, identifying novel disease-causing variants and understanding their impact on clinical phenotypes are important for evaluating disease progression and treatment.

The *SLC26A4* gene is located on the human autosome 7q22.3, contains 21 exons, and is closely associated with EVA occurrence and development. The pendrin protein encoded by *SLC26A4* plays a significant role in endolymphatic fluid resorption, acid-base balance, and proper function of the inner ear (Azaiez et al., 2007; Rozenfeld et al., 2011). Mutations in the *SLC26A4* gene can lead to functional defects in pendrin, causing abnormal anion transport and endocochlear potential in the inner ear. Mutations in the *SLC26A4* gene account for 13.73% of patients with hereditary hearing loss (Yuan et al., 2009), and the mutation rate of *SLC26A4* in Chinese simplex families with EVA or Mondini dysplasia is 97.9%, of which biallelic mutations account for 88.4% (Wang et al., 2007). According to domestic research, approximately 62%–88.4% of NSEVA cases are caused by double allelic mutations, and monoallelic mutations accounted for 7.4%–24% (Wu et al., 2010; Zhao et al., 2012). Other studies have shown that approximately two-thirds of Asian NSEVA patients have biallelic mutations in the *SLC26A4* gene (Wang et al., 2007; Wu et al., 2010; Miyagawa et al., 2014). Patients harboring different mutations may exhibit different clinical phenotypes. Therefore, identifying novel mutations and deepening our understanding of the relationship between the effects of these mutations and clinical phenotypes will be helpful for EVA diagnosis and treatment.

We report a case of profound sensorineural deafness in a boy with NSEVA. Novel compound heterozygous mutations, c.422T>C (p.Phe141Ser) and c.1523_1528delinsAC (p.Thr508Asn fs*3), in the *SLC26A4* gene were detected. This compound mutation caused a substitution of amino acids and premature truncation of the highly conserved domain of the pendrin protein, resulting in the impairment of pendrin in the mutant. This compound mutation may have been responsible for the profound sensorineural deafness phenotype observed in this patient.

## 2 Case presentation

A roughly 6-year-old boy presented to our hospital on 21 April 2019 due to “hearing loss for 2 year.” The patient was diagnosed with profound bilateral sensorineural deafness at the age of 4 years. The child could communicate normally after wearing hearing aids in both ears and undergoing speech rehabilitation training, but he could only hear voices within 3 meters; therefore, he was admitted for cochlear implantation surgery.

Physical examination showed a temperature of 36.8°C, pulse rate of 76 beats/min, respiratory rate of 20 beats/min, blood pressure of 110/75 mmHg, weight of 19 kg, and height of 114 cm. The child did not have mastoid tenderness or external ear deformity and had an unobstructed external auditory canal. The tympanic membrane was normal, and the tympanic curve was unobstructed. He had normal growth and development, was born full-term naturally, with a birth weight of 3,000 g, and passed the initial hearing screening 3 days after birth. He had a history of pneumonia but denied any exposure to ototoxic drugs, ear trauma, ear surgery, long-term noise exposure, early pregnancy infection, goiter, or diabetes mellitus. The parents were physically healthy and other members of the family had no hearing loss. The child underwent cochlear implantation in the left ear and follow-up showed good hearing test results.

## 3 Subjects and methods

### 3.1 Subjects and clinical examinations

A 6-year-old boy with profound sensorineural deafness was recruited and detailed history questioning, physical examinations, laboratory tests, and imaging examinations were performed. His parents were also enrolled in the present study. Blood-EDTA samples were obtained and subsequently used for DNA extraction and downstream sequencing analysis.

### 3.2 High-throughput sequencing

High-throughput sequencing was performed by the NGS platform of the BGISEQ (BGI-Shenzhen, China) using a hearing impairment panel. Briefly, genomic DNA was randomly fragmented and used for the library. The DNA in the coding region of the target genes and the adjacent splicing region was captured and enriched by the chip and sequenced through the BGISEQ platform. A total of 127 genetic deafness-related genes were analyzed.

### 3.3 PCR and Sanger sequencing

Genomic DNA was extracted from 1 mL EDTA-anticoagulated peripheral blood of all subjects using a TIANamp blood DNA kit (Tiangen, Beijing, China), according to the manufacturer’s protocol. DNA quality was quantified using a NanoDrop 2000c spectrophotometer (Thermo Fisher Scientific, Inc, Waltham, MA, USA). PCR was performed in a SimpliAmp™ PCR thermocycler (Applied Biosystems, United States) with a total volume of 20 μL, including 1×DNA Polymerase, 1×Taq Buffer, 2.5 mM of dNTP mixture, 0.5 µM of forward and reverse primers, and 50 ng of DNA template. The PCR conditions were as follows: 95°C for 5 min; 38 cycles at 95°C for 30 s, 60°C for 30 s, 72°C for 45 s, and a final extension at 72°C for 10 min. PCR products were assessed by 1.5% agarose gelelectrophoresis and directly sequenced with an ABI 3730xl DNA analyzer.

### 3.4 Mutation prediction and multiple sequence alignments

Three-dimensional structure molecular modeling of wild-type and mutant pendrin was carried out using the SWISS-MODEL (https://swissmodel.expasy.org/) program. The potential impact of the mutations was predicted by MutationTaster, Mutation Assessor, PolyPhen-2, and FATHMM software. The alignment of pendrin protein sequences in multiple species (obtained from the National Center for Biotechnology Information protein database, www.ncbi.nlm.nih.gov/protein) was performed using ClustalX2 software.

## 4 Results

### 4.1 Hearing, intelligence test, and imaging results

**FIGURE 1:**
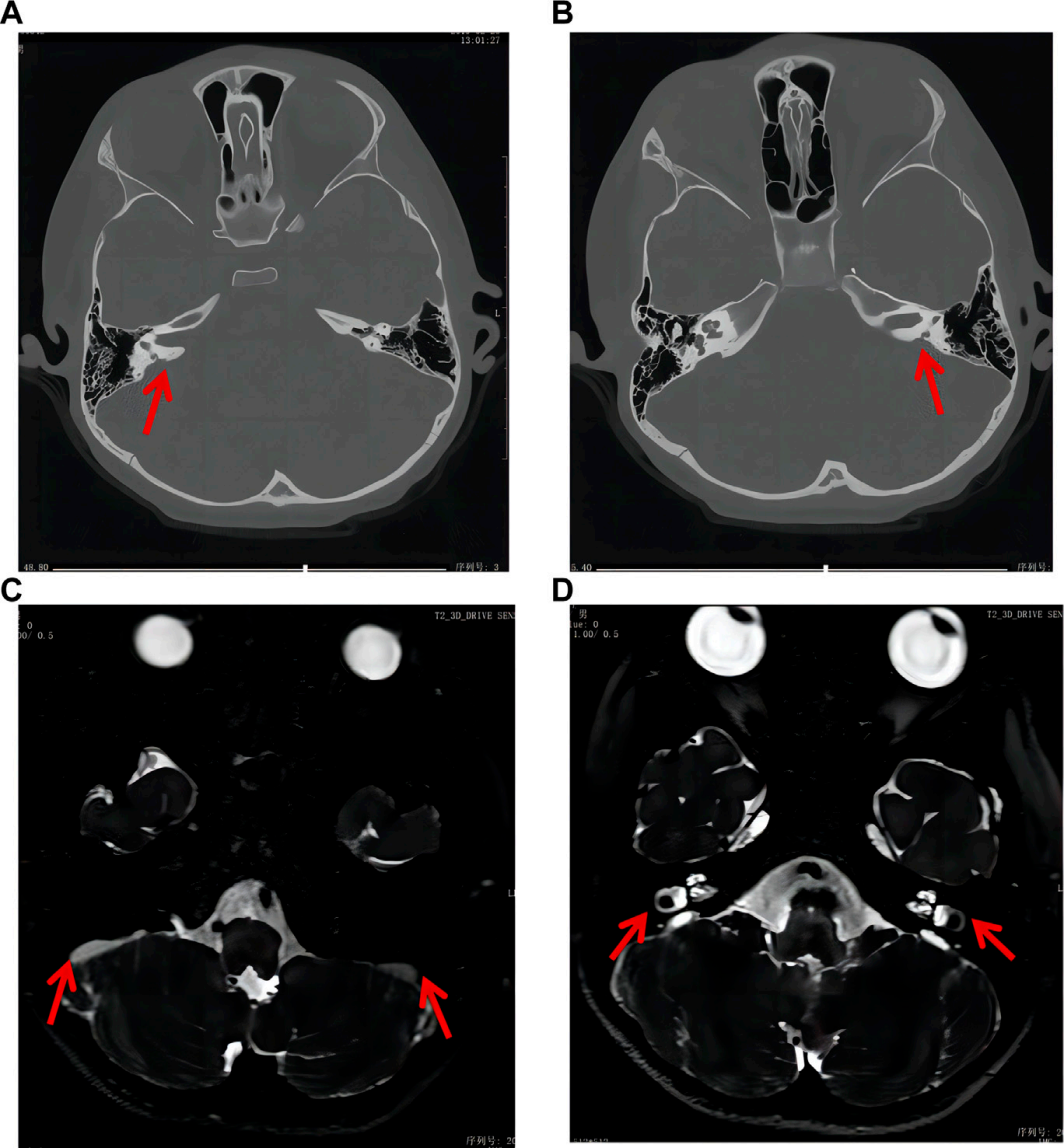
Craniocerebral CT and MRI results of the patient. **(A, B)** CT results indicated bilateral enlarged vestibular aqueduct (red arrow). **(C, D)** MRI results indicated bilateral endolymph sac effusion and enlarged vestibular aqueduct (red arrow).

The auditory brainstem response (ABR) test showed an increase in the response threshold and that the response wave was elicited at 100.0 dBnHL (reference value: 0–20 dBnHL) and 85 dBnHL for the left and right ears, respectively. The pure tone audiometry was significantly above the threshold value. Abnormal results were detected in binaural distortion product otoacoustic emission and acoustic reflex tests. Craniocerebral computed tomography (CT) and magnetic resonance imaging (MRI) showed bilateral enlarged vestibular aqueducts and endolymph sac effusion, respectively (Figure 1). The pattern of these symptoms was indicative of hearing loss. The patient had type-A tympanometry curves, suggesting normal middle ear function. The auditory behavior classification (CAP) and speech intelligibility classification (SIR) were tested and evaluated at level 1. Other physical and mental ability tests were normal, including the Griffiths intelligence, learning ability, and mental behavior development tests. The parents reported that the child achieved good hearing results after using the cochlear, and the pure tone audiometry results in the left ear were between 20 and 40 dB.

### 4.2 Chip capture high-throughput sequencing

**FIGURE 2:**
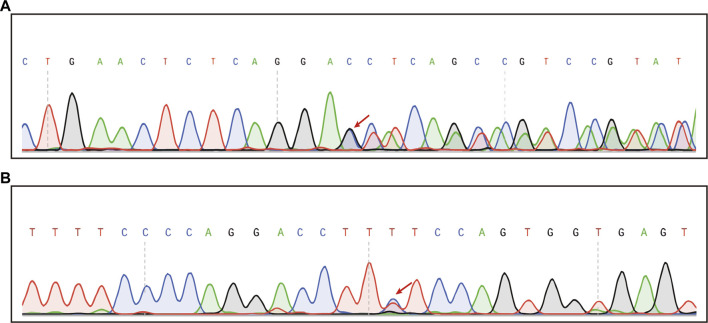
Chip capture high-throughput sequencing result in the patient. **(A)** Sequencing map of heterozygous c.1523_1528delinsAC mutation (Mutation start point is shown by red arrows). **(B)** Sequencing map of the heterozygous c.422T>C mutation (The mutation site is indicated by the red arrow).

Chip capture high-throughput sequencing was performed to analyze the mutation status of 127 genes associated with hereditary deafness in the proband, including GJB2, GJB6, *SLC26A4*, MT-RNR1, and MT-TS1 (Supplementary Table S1). The average sequencing depth was 369.66× and the coverage of the target region was 99.08%. The proportion of the average depth of the target area > 30× was 98.11%. The compound heterozygous mutations c.1523_ 1528delinsAC (p.Thr508Asnfs*3) and c.422T > C (p.Phe141Ser) in *SLC26A4* were identified in the proband (Figures 2A, B). The results were validated using Sanger sequencing. The boy’s mother and father were heterozygous for the c.1523_ 1528delinsAC and c.422T > C mutations, respectively. Notably, c.422T > C is known, whereas c.1523_ 1528delinsAC is a novel mutation that has not been previously reported.

### 4.3 Prediction of the mutations and multiple sequence alignments

**FIGURE 3:**
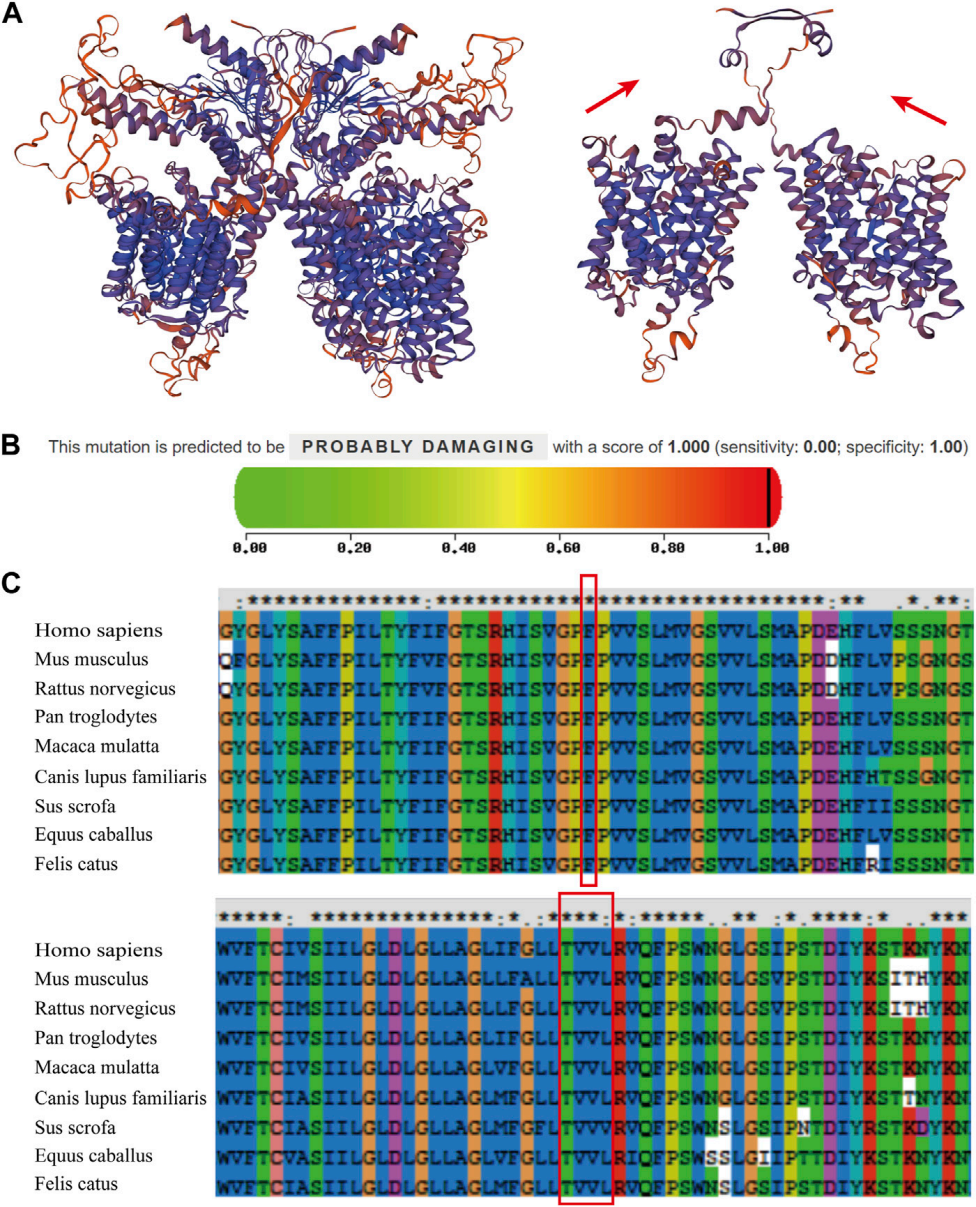
Prediction of the mutations and multiple sequence alignments for the patient’s two mutations. **(A)** The ribbon protein models of wild-type and c.1523_ 1528delinsAC mutant forms are displayed. The mutant protein exhibits a severely truncated form (red arrow). **(B)** The PolyPhen2 score was 1.000, predicted to be “PROBABLY DAMAGING” for c.422T > C. **(C)** Multiple sequence alignments revealed that these two mutations were located in the highly conserved amino acid region in different species (red boxes).

3D ribbon model *in silico* prediction was performed and the c.1523_ 1528delinsAC (p.Thr508Asnfs*3) mutation was predicted to cause a frameshift and emergence of an early stop codon, resulting in the production of a severely truncated pendrin protein (Figure 3A, red arrow). Mutation pathogenicity was predicted using four bioinformatics software packages: MutationTaster, Mutation Assessor, PolyPhen-2, and FATHMM. These results indicate that the c.422T > C mutation likely affected the structure and function of pendrin, and the c.1523_1528delinsAC mutation was suggested to be a disease-causing mutation (Table 1; Figure 3B). The mutated regions of multiple sequence alignments were also analyzed and the results indicated that the mutation sites were located in highly conserved pendrin regions in different species (Figure 3C, red box). These findings suggest that compound heterozygosity for the two mutations may be responsible for the severe hearing impairment in the proband.

**TABLE 1:** Mutation effect prediction using *in silico* software.

Prediction tool	Mutation site	Prediction result	Prediction score	Score range
MutationTaster	c.422T > C	Disease causing	1	0–1
PolyPhen2	c.422T > C	PROBABLY DAMAGING	1	0–1
Mutation Assessor	c.422T > C	Medium	2.295	−5.545–5.937
FATHMM	c.422T > C	DAMAGING	−2.78	—
FATHMM	c.1523_1528deli nsAC	DAMAGING	−3.27	—
MutationTaster	c.1523_1528deli nsAC	Disease causing	1	0–1

## 5 Discussion

Mutations in the *SLC26A4* gene, which encodes pendrin, are responsible for NSEVA (Li et al., 1998), an autosomal recessive disease (Abe et al., 1999). In the present study, two heterozygous mutations in *SLC26A4* were identified. The novel c.1523_1528delinsAC mutation has not been reported in the literature or in the ClinVar, PubMed, or HGMD databases. This insertion-deletion mutation causes premature termination of amino acid transcription, producing severely truncated proteins, and may have a strong impact on the structure and function of pendrin (Figure 3A). Another missense mutation, c.422T > C, has been previously reported (Chen et al., 2014); however, its pathogenicity was not clearly indicated. In this study, based on functional prediction by MutationTaster, Mutation Assessor, PolyPhen-2, and FATHMM, it was predicted to be “potentially damaging” or “damaging.” Based on the results of hearing tests and imaging examinations, the child was diagnosed with profound sensorineural deafness and bilateral vestibular aqueduct enlargement. However, the patient’s parents had normal clinical phenotypes. This implied that patients carrying these compound mutations exhibited a more severe phenotype than those carrying a single mutation.

The severity, laterality, and age of onset of NSEVA are highly variable (Honda and Griffth, 2022). SNHL is the primary clinical manifestation of NSEVA. Hearing loss in patients with NSEVA is reportedly acquired at birth or in early childhood, with bilateral involvement in 80% of cases, and is asymmetric, fluctuating, and progressive (Levenson et al., 1989; Govaerts et al., 1999; Zhao et al., 2018). Previous studies have noted that EVA patients with biallelic mutations mostly have severe-to-profound hearing loss and an earlier age of onset, a more fluctuating course, and more severe hearing loss compared with patients without pathogenic gene mutations (Zhao et al., 2019; Wu et al., 2022). Wang et al. (Wang et al., 2016) reported that two patients with severe SNHL and EVA harbored the compound heterozygous mutations c.1001+5G > C and c.919-2A > G3, in *SLC26A4*. The mother was a carrier of the c.919-2 A > G3 heterozygous mutation and developed sensorineural deafness but no EVA. The other three members of the family had c.1001+5G > C heterozygous mutations, with normal hearing and no EVA. Furthermore, Byun et al. (Byun et al., 2022) reported a family in which three children had c. 2168A > G and c.919-2A > G compound heterozygous mutations, and all passed newborn hearing screening at birth. However, they were found to have hearing loss and EVA at the ages of 4, 2, and nearly 5 years, respectively. The parents had heterozygous mutations, and two more dizygotic twin children in this family with c.919-2A > G heterozygous mutations in *SLC26A4* had no symptoms associated with EVA or hearing impairment. In this study, a child carrying two mutations in *SLC26A4* developed profound SNHL with EVA and endolymph sac effusion. The symptoms were similar to those reported in previous studies. Therefore, almost all biallelic patients develop severe sensorineural deafness and EVA; however, heterozygous carriers may exhibit no phenotypic abnormalities. A minority of hearing loss carriers may have other undiscovered mutations or environmental factors.

EVA is a characteristic manifestation of NSEVA and its pathogenesis is associated with mutations in the *SLC26A4* gene encoding for pendrin. Almost all (95.54%) patients with bilateral EVA carried biallelic *SLC26A4* mutations (Tian et al., 2021). The transmembrane protein pendrin encoded by *SLC26A4* acts as a nonspecific exchanger of anions such as Cl^−^ and I^−^ and bases such as HCO3^−^ and OH^−^ on the apical plasma membrane of epithelial cells (Honda and Griffth, 2022). Research has previously suggested that the reduced membrane expression and transport activity of mutant pendrin contribute to the pathogenesis of hearing loss in patients with EVA (Yuan et al., 2012). Mitochondria-rich cells in the endolymphatic sac express proton pump and pendrin proteins, transporting Cl^−^/HCO3^−^ and resolving HCO3^−^ upon H^+^ production. When pendrin function is impaired, H^+^ generation is blocked and the concentration of HCO3^−^ in mitochondria-rich cells increases, which affects Na^+^ reabsorption and causes dysfunction of fluid absorption, leading to expansion of the inner lymph sac, cochlea, and vestibular aqueduct. A cascade of events leads to the development of EVA and hearing loss (Kim and Wangemann, 2010; Honda et al., 2017; Xue and Chen, 2019).

Hearing aids and cochlear implants (CI) are the primary methods for hearing recovery in patients with EVA. Conventional hearing aids can provide good hearing rehabilitation in patients with mild-to-severe SNHL. However, CI is the preferred treatment as hearing loss progresses because hearing aids cannot meet their needs, and the early use of hearing aids may help patients better adapt to CI (Korver et al., 2017). A systematic review reported that post-CI patients showed clinical improvements in speech perception, auditory performance, language performance, and pure tone average (Benchetrit et al., 2022). Patel et al. also pointed out that patients with EVA who received CI showed significant improvements in pure tone average, speech acceptance threshold, and word score (Patel et al., 2018). Similarly, patients with *SLC26A4* mutations show good performance after cochlear implantation, and bilateral CI is superior to unilateral CI in terms of lexical outcome, speech perception, and sound localization (Nishio and Usami, 2017; Roh et al., 2017). Additionally, Na et al. showed that CI may reduce the frequency and degree of hearing fluctuations in patients with biallelic *SLC26A4* mutations (Na et al., 2021). In the present case, the hearing range of the child, who wore a hearing aid and underwent speech rehabilitation training, improved within 3 meters. To improve hearing, cochlear implantation was performed in the left ear, and later follow-up showed that he had a good auditory outcome; his pure tone audiometry results ear fluctuated between 20 and 40 dB, with significant improvement. Therefore, cochlear implantation has a significant benefit for EVA patients.

Previous studies, including this case study, have revealed several important points. First, early detection of hearing loss is beneficial for hearing development and the wellbeing of children (Korver et al., 2017), and neonatal deafness screening and hearing monitoring during growth are of great importance for the early diagnosis of sensorial deafness. Second, cochlear replacement can help improve hearing and can prevent language development disorders in children with EVA. Finally, parents with a family history of *SLC26A4* mutations should undergo genetic counseling, and prenatal diagnosis is feasible if necessary. In cases of significant hearing loss, especially congenital SNHL cases associated with inner ear malformations, *SLC26A4* variants and other common pathogen-related variants must be considered in molecular examinations (Roesch et al., 2018). High-throughput sequencing technologies offer significant advantages in the diagnosis of genetic diseases. Currently, high-throughput sequencing of fetal free DNA in maternal peripheral blood opens up a new method for the noninvasive prenatal diagnosis of hereditary deafness, which will further facilitate genetic counseling (He et al., 2022).

## 6 Conclusion

This study demonstrates that the novel mutation c.1523_1528delinsAC in compound heterozygosity with c.422T > C in the *SLC26A4* gene is likely to be the cause of NSEVA. Patients with double-site mutations exhibit severe sensorineural deafness and EVA. Early diagnosis, timely intervention, and genetic counseling are crucial for reducing disease progression and adverse effects. The findings of our study expand the mutational spectrum of *SLC26A4* associated with NSEVA and may be helpful for the prevention and early intervention of family based NSEVA.

## Data Availability

The datasets for this article are not publicly available due to concerns regarding participant/patient anonymity. Requests to access the datasets should be directed to the corresponding author.
