# Supplementary material for: Non-syndromic enlarged vestibular aqueduct caused by novel compound mutations of the SLC26A4 gene: a case report and literature review
Source: Front Genet. 2023 Sep 7;14:1240701. doi: 10.3389/fgene.2023.1240701 (PMC10512862; doi:10.3389/fgene.2023.1240701)
Supplement: Supplementary file 1 [file Table1.docx]

**Supplementary Table 1. High-throughput sequencing for 127 genes associated with hereditary deafness**.

| Autosomal recessive nonsyndromic hearing impairment | GJB2,GJB6,MYO7A,MYO15A,FOXI1,KCNJ10,SLC26A4,TMIE,TMC1, TMPRSS3,OTOF,CDH23,ATP2B2,GIPC3,STRC,OTOG,USH1C,TECTA,OTOA,PCDH15,RDX,GRXCR1,TRIOBP,CLDN14,MYO3A,DFNB31, ESRRB,ESPN,MYO6,GJA1,HGF,ILDR1,MARVELD2,DFNB59,SLC26A5,LRTOMT,LHFPL5,BSND,MSRB3,LOXHD1,TPRN,GPSM2,PTPRQ,SERPINB6,GJB3 |
| --- | --- |
| X-link hereditary hearing impairment | PRPS1,POU3F4,SMPX |
| Syndromic hearing impairment | SERAC1,PDSS1,FGFR3,FGFR1,FGFR2,PHEX,DLX5,TNFRSF11B, COL2A1,COL11A1,COL9A1,COL9A2,COL4A3,COL4A4,COL4A5, BSND,SOX9,PAX2,GATA3,SLC19A2,IGF1,PAX3,MITF,SNAI2,EDNRB,EDN3,SOX10,HOXA1,SOBP,EYA1,SIX5,SIX1,CHD7,SEMA3E,SMAD4,FGF3,TCOF1,PRRX1,GLI3,HOXA2,KCNQ1,KCNE1,CACNA1D,ALMS1,LRP2,TIMM8A,NDP,WFS1,OPA1,SLC4A11,MYO7A,USH1C,CDH23,PCDH15,USH1G,USH2A,GPR98,PDZD7,DFNB31,CLRN1,MTTK,MTTE,MTTL1,SLC26A4,KCNJ10,FOXI1 |
| Autosomal dominant nonsyndromic hearing impairment | ACTG1,CCDC50,CEACAM16,COCH,CRYM,DFNA5,DIABLO,DIAPH1,DSPP,EYA4,GJB2,GJB3,GJB6,GRHL2,KCNQ4,MIR96,MYH14,MYH9,MYO1A,MYO6,MYO7A,POU4F3,SIX1,SLC17A8,TECTA,TJP2,TMC1,WFS1,DIAPH3 |
| Maternally inherited hearing impairment | ACTG1,CCDC50,CEACAM16,COCH,CRYM,DFNA5,DIABLO,DIAPH1,DSPP,EYA4,GJB2,GJB3,GJB6,GRHL2,KCNQ4,MIR96,MYH14,MYH9,MYO1A,MYO6,MYO7A,POU4F3,SIX1,SLC17A8,TECTA,TJP2,TMC1,WFS1,DIAPH3 |
